# What's in a name?

**DOI:** 10.1308/rcsann.2024.0047

**Published:** 2024-05-01

**Authors:** B Rogers

**Figure:**
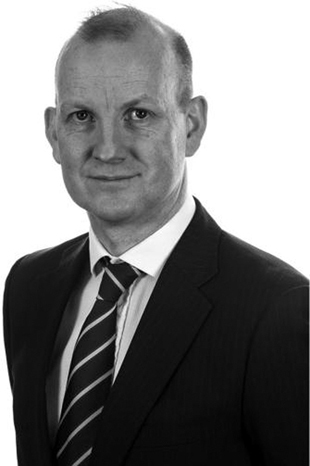
**B Rogers,** Editor-in-Chief *Annals* of the Royal College of Surgeons of England

The Royal College of Surgeons of England (RCS England) aims to support “..all our members, in all their diversity, to deliver excellence in everything they do.”^1^ The *Annals*, as a long standing component of the College, strives to reflect and augment the strategic aims of the College. Clinical research, and subsequent peer review process, has always been at the core of the profession and this publication continues this. Blending the twin goals of embracing the diversity of College membership with surgical research is the theme of this edition of the *Annals*.

The RCS England is rightly proud of its history and heritage but equally aware of the need for change and inclusivity. It is important to highlight that it is the Royal College of Surgeons *of* England, and not the Royal College of Surgeons *for* England. This choice of preposition is key – though often forgotten – with some assuming the College is too UK or London-centric. However, currently, the College membership has a large non-UK representation with 2229 surgical Fellows and 2261 surgical members. This diversity is a great strength of the College and should be emphasised.

The Global Surgical Frontiers (GSF) conference, hosted by the RCS England this June aims to bring together all stakeholders delivering surgical care in resource-limited settings.^2^ This forum will afford bilateral learning in understanding the challenges and the evidence-based solutions that exist. Furthermore, no one can claim to practice surgery in an environment that is not, to some extent, resource-limited.

Evidence-based global surgery solutions are rare, but the *Annals* provides a portal for such research. In a small way, this edition of the *Annals* highlights the global nature of this journal and wider College. This month, we have collated research that originates outside of the UK to demonstrate the geographic diversity inherent in surgical research. Whether this is a review of ‘Nutcracker Syndrome’ (Trinidad), a randomised control trial of negative pressure therapy following lower limb amputation (India) or a 15-year study of superficial femoral artery aneurysms (Italy). I am proud and delighted with the diversity of subject and author nationality this edition published.

Most *Annals* submissions originate from within the UK, but non-UK submissions are increasing, and this should be encouraged. I hope this edition helps promote further a broad geographical catchment area of published research by the RCS England.

Juliet asked Romeo “What's in a name?..” and so should we. Maybe we should pause the next time we read a letterhead/web page/email from the Royal College of Surgeons of England and consider the full meaning. Namely *of* England but *for* the wider global surgical profession.
